# Competence of health care providers on care of newborns at birth in a level-1 health facility in Yaoundé, Cameroon

**Published:** 2012-03-14

**Authors:** Francisca Monebenimp, Makudjou Tenefopa, Valere Mve Koh, Innocent Kago

**Affiliations:** 1 University Teaching Hospital of Yaoundé and Faculty of Medicine and Biomedical Sciences, University of Yaoundé I, Cameroon; 2 University Teaching Hospital of Yaoundé and Faculty of Medicine and Pharmacy, University of Douala, Cameroon; 3 Cameroon Pediatric Society, Yaoundé, Cameroon

**Keywords:** Neonatal care, competence, health care providers, health facility, Cameroon

## Abstract

**Introduction:**

This is an observational study which was carried out at a level one health facility in Yaoundé from June to July 2009. The aim was to evaluate the competence of health care providers towards newborns’ care at birth

**Methods:**

Ten health care providers took care of three hundred and thirty-five pregnant women who were enrolled for the study after informed verbal consent in the delivery room.

**Results:**

Out of 340 offspring delivered and taken care of, 179 (52.6%) were male and 161 (47.4%) were female. Only two out of ten health workers had a WHO Essential Newborn Care (ENC) training. None of them had received any refresher course for the past two years. The mean gestational age of women was 39.5±3.5 weeks. Resuscitation was carried out on 21 (6.2%) of the newborns including 7 (33.3%) who had birth asphyxia. Health care providers scored 100% in performing the following tasks: warming up the baby, applying eye drops, injecting vitamin K, identifying the neonate, searching for any apparent life threatening congenital malformations, preventing for infection after procedures and initiating breastfeeding. The score was 24% at neonatal resuscitation tasks. Low level of education was associated with poor competence on applying ENC tasks (p<0.001). Lack of WHO ENC training was associated with poor competence on ENC tasks (p<0.001) and poor skills on resuscitation (p=0.03).

**Conclusion:**

There is a need to reinforce the capacity of health care providers by training in WHO ENC course with emphasis on providing skills on resuscitation in order to reduce the burden of neonatal intrapartum-related deaths.

## Introduction

About 99% of the four million neonatal deaths per year occur in low income countries [1]. Hospitals in many developing countries appear not well prepared to ensure newborn survival because of insufficient staff, poor hygienic conditions and organization to support the provision of care [2]. Several observations reveal that facility-based basic resuscitation may avert 30% of intrapartum-related neonatal deaths [3]. Several trials have shown that health workers in some countries can perform neonatal resuscitation with an estimated effect of 20% reduction of these deaths [3]. The World Health Organization Essential Newborn Care (WHO ENC) course has set minimum standards for training midwives in this domain. It has been shown that in facilities where midwives have received this training there has been a decrease in early neonatal mortality rates [4]. In Cameroon, the neonatal mortality rate was 40 per 1000 live births in 2004 [5]. WHO ENC implementation had started slowly in some areas and it is not known how health personnel perform these specific activities. In this study we want to assess the competence of health workers towards newborns’ care at birth in a level-one health facility to know what the gaps are so as to reinforce their capacity.

## Methods

### Study setting

This is an observational study carried out from June to July 2009 at the Centre for social animation and Health (CASS) of Nkoldongo, Yaoundé. This is a catholic health facility that reported a large recruitment of pregnant in 2008 with 3733 deliveries [6]. Women coming in this centre for antenatal care (ANC) visits and presenting with high risk pregnancies are immediately referred to a higher level health facility for follow up. The facility has a labor room, a delivery room and a postpartum ward having respectively three, two and 33 beds. There are 10 health care providers who work permanently in the delivery room grouped in three teams with an eight hour shift schedule. The health workers were all registered nurses but one that was an aid nurse at the beginning of her carrier had a long experience in nursing. There is a pediatrician who comes twice a week for neonatal visits and two nurses that coordinate activities of the unit. The delivery room had a radiant warmer, source of oxygen, bag and mask, and consumables for infection prevention.

**Data collection** A pre-validated data collecting form was used to gather information from health workers and from mothers’ obstetrical and gynaecological files. This was on health workers’ level of education, refresher course status and training on WHO ENC. The level of education was low when a health worker ended at primary school, middle or high when he or she reached respectively secondary or university level. For mothers, the gestational age, history of pregnancy related to the number of antenatal care visits, intermittent treatment for malaria and HIV serology were recorded. Competence of health care providers was assessed by direct observation of how the newborn was handled. The only observer, an intern at the end of medical training, who had a training on WHO ENC noted whether the health worker identified the newborn at delivery, searched for any congenital malformations mostly those that were apparent and life threatening such as imperforated anus, choanal atresia when there was difficult breathing and no passage while introducing a 4F nasograstric tube in the nostrils, abdominal wall hernia, to cite only these. Essential newborn care management, resuscitation in case of difficulty in initiating breathing and prevention of infection were also assessed as described below. It should be mentioned that the health workers were aware of the fact that the observer was collecting data, without knowing specifically which one. National guidelines adapted from WHO recommendations [7] were used. There were 19 items distributed as follow: **Identification of neonates** which included recording of the gestational age, weight, sex and the Apgar score at 1 and 5 minutes. The search of any congenital malformations was included. **ENC tasks** were cord care (cutting the cord with sterile material and tying it with sterile thread), fight against hypothermia (drying up the newborn, putting him wrapped close to mother or under the radiant warmer) and injection of vitamin K, eye care (wiping the eyes with sterile gauze before applying eye drops and not wiping again) and breast feeding were part of the assessment. **Resuscitation** had several steps but the focus was on checking the equipment, positioning of the head of the neonate (neutral position), aspiration, positioning of the mask (nose and chin under the mask) and ventilation (making sure the chest was moving up when insufflating air). **Prevention of infection** included hand washing, decontamination and disinfection of the used equipment. Scoring was made by assigning 1 point each time a task was applied. For tasks that needed more skills such as resuscitation, 1 point was given when the execution of the task was incomplete, 2 points when it was made correctly but the steps were not adequately followed and 3 points when the task was mastered correctly and precisely. Identification of neonates had 6 points, ENC tasks 7, resuscitation 17 and prevention of infection had 3 points. For easy calculation, scores were presented in percentage. For each subset, the mean score for a health worker was calculated and the total score was an aggregate or sum of the subset scores.

It was assumed that 32% of newborns will be resuscitated it was assumed that 32% of newborns will resuscitated as found by Takpara et al in Benin [8], with an error of 5%, the sample size calculated was 340. Pregnant women, after giving their verbal informed consent were enrolled consecutively in the study. At the end, each health worker had to take care of 34 newborns. To make it simple, the sample size was divided among the 10 health care providers. When a health care provider reached the number of 34, the observation was discontinued for he or she. Data were analysed using Epi Info software version 3.5.1 and SPSS version 12.0. Chi square test was used to compare proportions and the level of significance was set at 0.05. A univariate analysis was conducted to identify potential variables associated with competence. ANOVAs test was also used to compare mean scores.

## Results

During the two months period, the ten health care providers recruited 335 pregnant women who delivered 340 live newborns. Among these newborns, 179 (52.6%) were male. Table 1 shows the demographic characteristics of the health workers. None of them had received any refresher course in the past two years and only 2 (20%) had training on WHO ENC. Nine (90%) health providers were female and 9 (90%) had a college level of education. Concerning antenatal history (Table 2), among the enrolled pregnant women, 3 (0.9%) did not attend any antenatal clinic and 130 (38.8%) of women had less than four antenatal consultations. At least two echographies were completed by 233 (69.5%) women. The mean gestational age was 39.5±3.5 weeks. Only 158/335 (47.2%) took adequate malaria prophylaxis, 41 (12.1%) had malaria in pregnancy and 21 (6.3%) had a positive test for Human Immunodeficiency Virus. Looking at intapartum follow up, 4 (1.2%) women had a prolonged labor. 25 (7.4%) had premature rupture of membranes, 17 (5%) had meconium stained amniotic fluid. Cord around the neck was noticed in 6 (1.8%) newborns. The Apgar score was below 5 at one minute for 21(6.2%) neonates and at five minutes for 7 (2.46%) newborns. Taking in to consideration newborns who did not breathe at birth, 7 (33.3%) had birth asphyxia and out of them 4 (57.1%) newborns had a poor status including 2 (50%) who died in the delivery room. The remaining 2 (50%) were referred for proper management at a higher level facility, the University teaching hospital (UTH) of Yaoundé.

**Table 1:** Demographic characteristics of the health care providers in a level-one health facility, CASS, June to July 2009

Parameters	frequency	Percentage
Female sex	09	90
Married status	10	100
Primary level of education	01	10
College level of education	09	90
Training in WHO ENC	02	20
Refresher course in the past two years	0	0

CASS: Centre for Social Animation and Health; ENC: Ante Natal Care

**Table 2:** Distribution of pregnant women in relation with antenatal and intra partum history in the level-one health facility, CASS (Centre for Social Animation and Health), June to July 2009

Parameters	frequency	Percentage
**Prenatal history**		
Gestational age < 37 weeks	25	7.4
Gestational ^3^ 42 weeks	23	6.8
At least 4 ANC	205	38.8
None obstetrical echography	96	28.7
At least 2 echographies	233	69.5
No antimalarial prophylaxis	20	06
One dose of intermittent treatment for malaria	118	35.2
Two doses of intermittent treatment of malaria	158	47.5
HIV Positive serology	21	6.3
No HIV testing	10	03
Malaria in pregnancy	41	12.1
Multiple pregnancy	10	2.9
**Intra partum history**		
Cord around the neck	06	1.8
Premature rupture of membranes	25	7.4
Prolonged labor	04	1.2
Meconium stained amniotic fluid	17	05

ANC: Ante Natal care

### **Evaluation of completed tasks** (Table 3)

Identification of neonates was carried out by all the health care providers. All the newborns 340 (100%) had the gestational age and sex recorded. So were the weight and the evaluation of Apgar score at 1 and 5 minutes. The search for congenital malformations was systematic for all the babies. Therefore, health workers had 100% score.

**Table 3:** Health care providers’ completed tasks by domain assessed in a level-one health facility, CASS (Centre for Social Animation and Health), June to July 2009

Parameters	Frequency of completed tasks by the health workers (%) n=340	Scores %
**Identification of neonates**		100
Gestational age	340 (100)	
Weight	340 (100)	
Sex	340 (100)	
Apgar score at 1minute	340 (100)	
Apgar score at 5 minutes	340 (100)	
Search: congenital malformations	340 (100)	
**ENC tasks**		100
Cord care	340 (100)	
Warming up	340 (100)	
Vitamin K injection	340 (100)	
Eye care	340 (100)	
Breast feeding*	319 (93.8)	
Resuscitation*		24
Equipment verification	2 (9.5)	
Positioning of new-born	2 (9.5)	
Aspiration	21 (100)	
Positioning of mask	2 (9.5)	
Ventilation	2 (9.5)	
**Prevention of infection**		100
Hand washing	340 (100)	
Decontamination of equipment	340 (100)	
Disinfection of equipment	340 (100)	

* Newborns resuscitated; ENC: Ante natal Care

ENC tasks were undertaken by all the health workers on all newborns. Cutting the cord was properly conducted. Warming up the newborn was done. Baby was dried up then wrapped and put on mother’s abdomen or under the radiant warmer while taking care of the mother. The score was 100%. All the newborns had vitamin K injection giving 100% score. Eye drops were administered to all babies but the eye care technique needed to be improved in 274 (80.6%) of cases because wiping with gauze before applying drops was not fulfilled. Newborns who did not experience respiratory distress 319 (93.8%) were breastfed in between one hour time after delivery. So, health workers scored 100%.

Resuscitation was carried out on 21 (6.2%) cases that did not breathe at birth. Checking the functionality of the bag and mask was done 2/21 (9.5%) times so as the positioning of the head of the newborn and the mask on the face. Ventilation was conducted in20/21 (95.2%) children that needed resuscitation. All the newborns were aspirated. Cardiac massage was performed on 20/21 (95.2%) newborns and the technique needed to be improved even if this was not part of the assessment. The health workers had 24% of completed tasks.

Prevention of infection which included hand washing, decontamination and disinfection of equipment was completed at 100% score by health care providers.

### Evaluation of competence mean scores (Table 4)

In the domain of identification of neonates, health workers had a mean score of 5.01±0.07 points out of 6. At ENC tasks, the mean score was 5.19±0.40 out of 7 points. For Resuscitation they scored 6.05±0.22 points out of 17. At the domain of prevention of infection their score was 3±0.0 out of 3 points. Factors associated with competence displayed on Table 5 showed that having a higher level of education was associated with good performance in applying ENC Tasks (P < 0.001) and in Table 6, not having a training was associated with poor competence in performing ENC tasks (P < 0.001) and resuscitating a newborn (p = 0.03).

**Table 4:** Mean scores competence of health care providers by domain assessed in a level-one health facility, CASS (Centre for Social Animation and Health), June to July 2009

Domain	Health workers mean scores (standard deviation)	Maximum score	P value
Identification of neonates	5.01 (0.07)	6	0.48
ENC tasks	5.19 (0.04)	7	<0.001
Resuscitation	6.05 (0.22)	17	0.001
Prevention of infection	3	3	–

ENC: Ante Natal Care

**Table 5:** Factors associated with competence using mean scores of health workers by level of education in a level-one health facility, CASS, June to July 2009

Parameters	Level of education		P value
	Primary	Secondary	
Identification of neonate	5.03 (0.03)	5.00 (0.0)	0.06
ENC Tasks	5.00 (0.0)	5.21 (0.42)	< 0.001
Resuscitation	6.00 (0.0)	6.05 (0.23)	0.69
Prevention of infection	3.00 (0.0)	3.00 (0.0)	–

P< 0.05 is significant; ENC: Ante Natal Care

**Table 6:** Factors associated with competence using mean scores of health workers by training in Essential Newborn Care in a level-one health facility, CASS (Centre for Social Animation and Health), June to July 2009

Parameters	Training in ENC		P value
	Trained	No training	
Identification of neonate	5.00 (0.0)	5.01 (0.08)	0.47
ENC Tasks	5.91 (0.28)	5.01 (0.13)	< 0.001
Resuscitation	6.25 (0.50)	6.00 (0.0)	0.03
Prevention of infection	3.00 (0.0)	3.00 (0.0)	–

P< 0.05 is significant; ANC: Ante Natal Care

## Discussion

This observational study conducted at a level one health facility enrolled 10 health workers that took care of 340 newborns in the delivery room. It has been shown that most of the health workers were female as noticed in this profession in many countries [9,10]. Only 02 (20%) of health care workers had WHO ENC training course. This finding gave the magnitude of the gap in trained health practitioners in this domain. The picture was almost similar to the one in other settings in developing countries [11]. Working on neonatal asphyxia, Sidibe et al revealed the importance of training health workers acting in delivery room [12].

The competence of health workers was good as the scores were respectively 100% in the domain of neonates’ identification, 100% for ENC tasks and 100% in the area of prevention of infection. This level of competence observed was surprising as we noticed that only 2 (20%) health workers had WHO ENC training course and none of them had a refresher course for the past two years. The number of years in the field and the real profile of the health worker could have influence this results even though, it has been shown that written and performance scores decreased significantly six months after training of health workers [13]. The study did not address this issues or the level of qualification of this health personnel. Health workers were not competent at resuscitating newborns who did not breathe at birth as they scored 24% because of lack of skills and training. This was on line with the findings in Pakistan where health care providers performed poorly and only 50% were able to demonstrate steps of immediate newborn care [14]. The fact that the heath care provider knew that he /she was observed could have influenced the way care of the newborn was conducted. But to minimize this, the observer was looking by the glass window in between the labor room and delivery room. Birth asphyxia was a big challenge for these health care providers as 2 (50%) of the newborns that had a poor status died in the delivery room. The transferred newborns died on the same day of referral to the UTH. The absence of good transportation referral system in the country might be an associate factor leading to death for these two newborns (personal communication). The prevalence of premature deliveries was 7.4% which was less than 11.11 % found in the same period at the UTH of Yaoundé [15]. This could reflect the quality of counseling during antenatal consultations as the awareness might be enhanced on self referral to a higher level health facility for better management of prematurity. That could also be an explanation for the low rate of birth asphyxia compared to the one of 35.1% noticed at this referral hospital in town [15]. All these deaths could be averted if effective basic neonatal resuscitation was conducted [4,16] presumably as the two health workers who had a former WHO ENC training were not the one who took care of these asphyxiated babies. The study demonstrated that lack of WHO ENC training of the health care providers was significantly associated with poor competence on ENC tasks and poor skills on resuscitation. This could explain the high number of newborns death encountered. This supported the finding that ENC training of health workers was significantly associated with a decrease in early neonatal mortality [4] in particular ENC training of midwives in low risk clinics was significantly associated with decreased 7-day mortality rates (relative risk: 0.59 (95% confidence interval: 0.48–0.77); P < .001) [17]. A clinical trial on the implementation of WHO ENC conducted in six countries Argentina, Democratic Republic of Congo, Guatemala, India, Pakistan, and Zambia revealed that apart from the significant reduction of stillbirths, there was no significant reduction from baseline in the rate of perinatal and neonatal death [18]. It seemed important to address the issue that these countries had an ongoing ENC program that could explain why the effect could not be seen as pointed out by the authors, henceforth in our setting where this approach is not spread nationwide, the effect of such a program would be more cost-effective as demonstrated in South East Central and Southern East Africa [19]. One might point that this level one health facility situated in the capital city had the advantage of being supervised by a consultant pediatrician twice a week. That could have influenced the knowledge and the competence of health care providers either way [20]. Misclassification bias could be noticed as far as scoring was concerned mostly in resuscitation domain. Cardiac massage was not part of the assessment but most of the newborns who did not breath at birth got it even though all of them might not have been in need. This could have happened because of the persistence of hypoxia due to poor ventilation technique [3,21]. Nevertheless, these results could not be easily generalized to other level one health facilities at country level because the capital city location also, the small sample size was a limitation for the inferences made.

## Conclusion

This study showed that the majority of health workers had no WHO ENC training. Health care providers were competent at providing basic essential newborn care. They lacked skills for proper handling of newborns that do not breathe at birth. There is an urgent need to reinforce the capacity of health care providers in this health facility as far as resuscitation is concerned and this could be extended to the overall country in order to meet MDG 5 by the year 2015.
